# Systematic Review, Meta-Analysis, and Population Study to Determine the Biologic Sex Ratio in Dilated Cardiomyopathy

**DOI:** 10.1161/CIRCULATIONAHA.124.070872

**Published:** 2025-02-03

**Authors:** Natalie Bergan, Ishika Prachee, Lara Curran, Kathryn A. McGurk, Chang Lu, Antonio de Marvao, Wenjia Bai, Brian P. Halliday, John Gregson, Declan P. O’Regan, James S. Ware, Upasana Tayal

**Affiliations:** 1 National Heart Lung Institute, Imperial College London, UK (N.B., L.C., K.A.M., B.P.H., J.S.W., U.T.); 2 Biomedical Image Analysis Group, Department of Computing, London, UK (W.B.); 3 Department of Brain Sciences, London, UK (W.B.); 4 Institute of Clinical Sciences, London, UK (D.P.O.); 5 Imperial College Healthcare NHS Trust, London, UK (J.S.W.).; 6 MRC Laboratory of Medical Sciences, London, UK (K.A.M., C.L., A.d.M., D.P.O., J.S.W.).; 7 Royal Brompton & Harefield Hospitals, Guy’s and St Thomas’ NHS Foundation Trust, London, UK (I.P., B.P.H., J.S.W., U.T.).; 8 London School of Hygiene and Tropical Medicine, UK (J.G.).; 9 Department of Women and Children’s Health and British Heart Foundation Centre of Research Excellence, School of Cardiovascular and Metabolic Medicine and Sciences, King’s College London, UK (A.d.M.).; 10 The George Institute for Global Health, UK (U.T.).

**Keywords:** cardiomyopathy, dilated, penetrance, sex

## Abstract

**BACKGROUND:**

Dilated cardiomyopathy (DCM) appears to be diagnosed twice as often in male than in female patients. This could be attributed to underdiagnosis in female patients or sex differences in susceptibility. Up to 30% of cases have an autosomal dominant monogenic cause, where equal sex prevalence would be expected. The aim of this systematic review, meta-analysis, and population study was to assess the sex ratio in patients with DCM, stratified by genetic status, and evaluate whether this is influenced by diagnostic bias.

**METHODS:**

A literature search identified DCM patient cohorts with discernible sex ratios. Exclusion criteria were studies with a small (n<100), pediatric, or peripartum population. Meta-analysis and metaregression compared the proportion of female participants for an overall DCM cohort and the following subtypes: all genetic DCM, individual selected DCM genes (*TTN* and *LMNA*), and gene-elusive DCM. Population DCM sex ratios generated from diagnostic codes were also compared with those from sex-specific means using the UK Biobank imaging cohort; this established ICD coded, novel imaging-first, and genotype first determined sex ratios.

**RESULTS:**

A total of 99 studies, with 37 525 participants, were included. The overall DCM cohort had a 0.30 female proportion (95% CI, 0.28–0.32), corresponding to a male:female ratio (M:F) of 2.38:1. This was similar to patients with an identified DCM variant (0.31 [95% CI, 0.26–0.36]; M:F 2.22:1; *P*=0.56). There was also no significant difference when compared with patients with gene-elusive DCM (0.30 [95% CI, 0.24–0.37]; M:F 2.29:1; *P*=0.81). Furthermore, the ratio within autosomal dominant gene variants was not significantly different for *TTN* (0.28 [95% CI, 0.22–0.36]; M:F 2.51:1; *P*=0.82) or *LMNA* (0.35 [95% CI, 0.27–0.44]; M:F 1.84:1; *P*=0.41). Overall, the sex ratio for DCM in people with disease attributed to autosomal dominant gene variants was similar to the all-cause group (0.34 [95% CI, 0.28–0.40]; M:F 1.98:1; *P*=0.19). In the UK Biobank (n=47 549), DCM defined by International Classification of Diseases, 10th revision, coding had 4.5:1 M:F. However, implementing sex-specific imaging-first and genotype-first diagnostic approaches changed this to 1.7:1 and 2.3:1, respectively.

**CONCLUSIONS:**

This study demonstrates that DCM is twice as prevalent in male patients. This was partially mitigated by implementing sex-specific DCM diagnostic criteria. The persistent male excess in genotype-positive patients with an equally prevalent genetic risk suggests additional genetic or environmental drivers for sex-biased penetrance.

**REGISTRATION:**

URL: https://www.crd.york.ac.uk/prospero; Unique identifier: CRD42023451944.

**Clinical Perspective**

**What Is New?**

- Among individuals with autosomal dominant inheritance of dilated cardiomyopathy (DCM), an equal sex distribution is expected. This study systematically evaluated the sex ratio in DCM (n=37 525), finding that all-cause, monogenic, and gene-elusive DCM are characterized by a ≈2:1 male to female ratio.
- The sex imbalance partially corrects on implementing novel sex-specific diagnostic criteria (UK Biobank [n=47 549]), suggesting that DCM may not be adequately detected in female patients, leading to underdiagnosis.
- The sex imbalance does not fully correct, suggesting that male patients are either more susceptible to DCM due to genetic or environmental factors or that female patients are protected in some way.

**What Are the Clinical Implications?**

- Increased clinical vigilance may be required to diagnose DCM in female patients across the disease spectrum.
- The application of sex-specific DCM diagnostic criteria should be evaluated.
- Genetic, reproductive, and environmental factors contributing to differences in disease risk in male and female patients should be explored.

Dilated cardiomyopathy (DCM) is a primary cardiomyopathy clinically defined by the presence of left or biventricular dilation and contractile dysfunction that cannot be explained by other causes, such as coronary artery disease or abnormal loading conditions.^1,2^ DCM has a prevalence of ≈1 in every 250 people, with an estimated overall male preponderance of 2.5:1.^1,3–5^ The development of DCM has been attributed to both genetic and acquired factors, with ≈20% to 30% of cases having an identified monogenic cause.^2,6,7^ The genetic pathogenesis of DCM is complex, as evident by the diversity of implicated genes and the range of observed inheritance patterns.^6,8^ In addition, DCM-associated genetic variants exhibit age-related and incomplete penetrance, with most monogenic forms of DCM being highly penetrant by late adulthood in family studies.^6,7^ Recent gene curation efforts for nonsyndromic DCM have defined 12 genes with robust evidence of monogenic disease causation (Table S1).^2,8,9^ Each of these genes exhibits an autosomal dominant (AD) inheritance pattern, which is primarily observed in adult-onset forms of DCM.^6,9^

Both genetic and nongenetic forms of DCM have been reported to occur more commonly in male patients.^4,10^ Among all-cause DCM, this could plausibly be attributed to biases in diagnosis and nonbiologic factors, leading to underdiagnosis in female patients. However, a similar male preponderance has been observed within AD monogenic DCM, where the prevalence of predisposition to the condition stratified by sex (sex ratio) would be expected to be 1:1 male to female, assuming equal penetrance in male and female individuals.^11^ For patients with a titin-truncating variant (*TTN*tv)—the most common DCM-associated variant—the male:female ratio (M:F) has been reported to be as high as 3:1.^9,10^ Another study encompassing adult-onset variant-positive DCM described an M:F of 1.7:1.^4^ Although the incomplete penetrance of DCM variants could explain this observation, a sex ratio closer to 1:1 would have been expected given the equal inheritance risk of AD DCM variants in both sexes.

The generalizability of these and other previous studies is restricted by their small sample sizes and the limited description of gene-level effects, indicating the need for a systematic assessment of the sex ratio in genetic, gene-elusive, and all-cause DCM. If the male preponderance exists even in AD genetic forms of DCM, then investigation into the drivers of sex-specific penetrance would be urgently warranted. We therefore conducted a systematic review and meta-analysis to systematically analyze evidence from DCM cohort studies to assess the M:F across DCM subtypes. Because DCM is diagnosed in a sex-independent manner, we also investigated whether the observed sex ratios could be influenced by diagnostic bias.

## METHODS

### Literature Search Strategy

**Figure 1.**
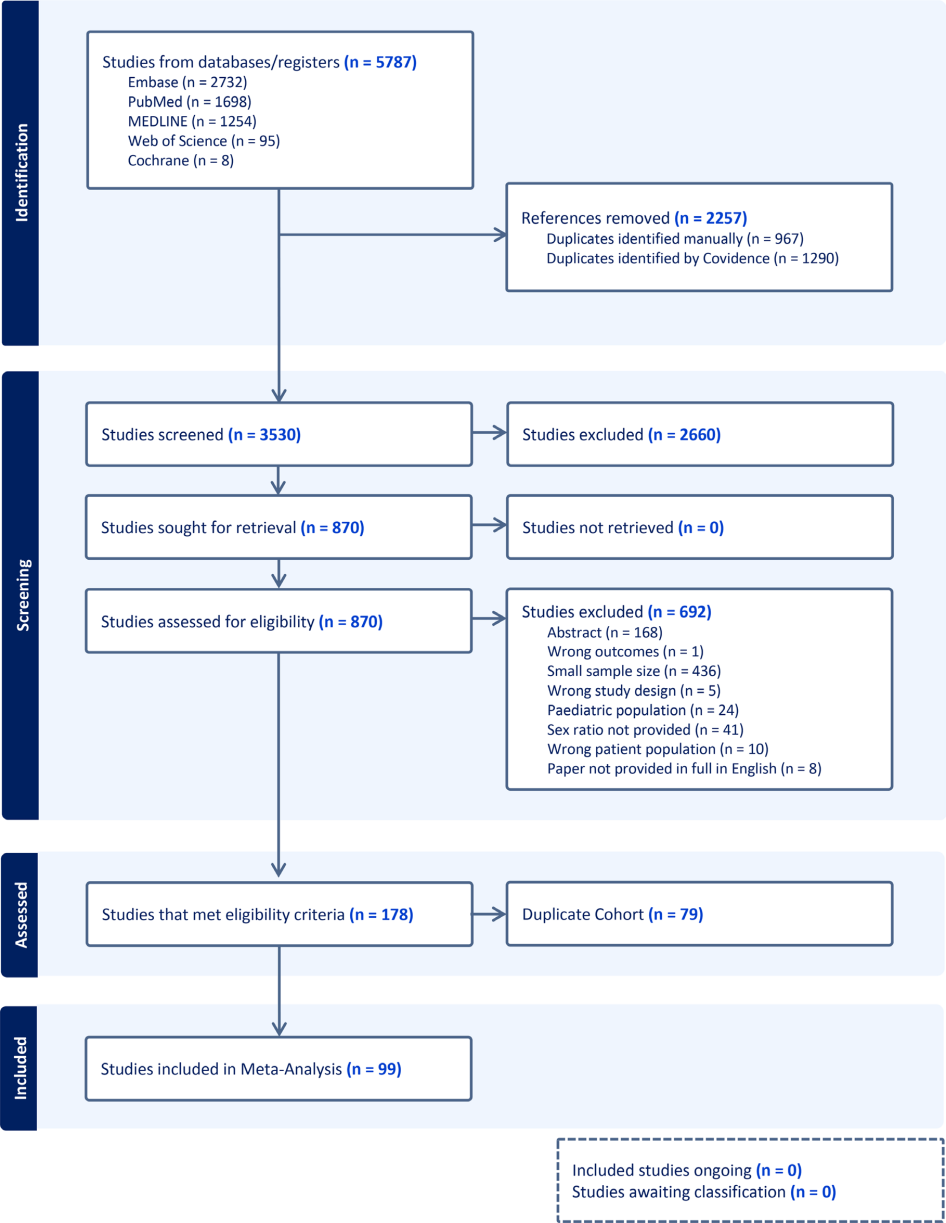
**Preferred reporting items for systematic reviews and meta-analyses flow diagram.** Flow diagram detailing the number of studies that were included in each step of the literature search screening,^13^ and the origin of the included studies, the number of duplicates removed, and the reason behind exclusion from this study. The figure was generated using Covidence.^150^

The literature review was performed in accordance with Preferred Reporting Items for Systematic Reviews and Meta-Analyses guidelines (Figure 1, Figures S1 and S2, and Methods in the Supplemental Material).^13^ The review question was as follows: How does the M:F of prevalent DCM compare with subgroups defined by the presence or absence of pathogenic variants within the defined DCM-associated genes? To address this, we identified articles containing DCM patient cohorts by focusing on 3 subject areas: DCM, the 12 genes with strong or definitive evidence for causing DCM (Table S1), and the type of DCM onset.^9^

The final systematic search was performed on June 26, 2023. Search results were extracted from PubMed, MEDLINE Ovid, EMBASE, the Cochrane Central Register of Controlled Trials, and Web of Science. For each search both key words and Medical Subject Heading terms were included. The interest in genetic DCM was defined using the term “gene*” and by listing the 12 genes given a “definitive” or “strong” association by the ClinGen Gene Curation Expert Panel.^9,14^ In line with this, we based our definition of genetic DCM on the genes in which there is high evidence that pathogenic variants clearly cause DCM (Table S1). For type of DCM onset, we specified DCM studies that discussed adult, late-onset, and nonsyndromic forms. This analysis focuses on sex, referring to the different biologic and physiologic characteristics of male and female individuals, and not gender, which refers to the socially constructed characteristics of women and men.^12^ The full search strategies for each of the searched online databases can be seen in the Supplemental Material (Methods and Table S2).

### Study Eligibility and Selection

Filtering of articles for relevance was carried out by 2 independent investigators (N.B. and I.P.) and divided into title and abstract screening and full-text screening. Studies were excluded if they (1) were identified to be unrelated to DCM, (2) described in vitro functional studies, (3) included pediatric, syndromic, X-linked, mitochondrial, or autosomal recessive forms of DCM, (4) included ischemic DCM or peripartum cardiomyopathy,^15^ (5) described DCM patient cohorts <100 participants, (6) did not provide male and female patient counts, (7) included a majority of patients <18 years of age, (8) did not include a clinical diagnosis of DCM, or (9) were not available in the English language.

Articles that met the eligibility criteria were assessed for cohort duplication, which was defined by the cohorts deriving from the same source with overlapping years of data collection. If the source of a cohort was unclear, the hospital or university of the first author was used as a substitute. When duplicate cohort sources were identified, the article that described a genetic DCM cohort progressed and all others were excluded. If no genetic DCM cohort was described, the article with the largest cohort was chosen. If the cohort size was the same, the article published most recently was progressed. If studies described multiple DCM cohorts from different sources, the cohorts were differentiated and all nonduplicated data were extracted.

### Data Extraction and Quality Assessment

Quality assessment and data extraction were performed in Covidence using customized templates (see Supplemental Material). The extracted data were then categorized into 3 cohorts: all participants (general DCM), those who had a pathogenic variant within 1 of the 12 defined genes (genetic DCM), and those who received genetic testing but did not have a pathogenic variant identified (gene-elusive DCM). A patient was determined to have gene-elusive (nonmonogenic) DCM only if they had genetic testing, had been tested for variants in at least 9 of the 12 ClinGen genes, and no pathogenic or likely pathogenic variants had been identified.^9^

The quality assessment template was based on the Newcastle-Ottawa Quality Assessment Scale for case–control and cohort studies and the Q-Genie tool.^16,17^ Risks of bias relating to the study’s participants, specifically assessing bias caused by genetic variant classification, retrospective enrollment (to reflect ascertainment bias from a retrospective cohort design), inclusion criteria, and selective reporting, were all assessed and given a risk rating of low, some concerns, or high. Bias related to variant classification was assessed by comparing each study’s reported method with the American College of Medical Genetics and Genomics classification.^18^ Inclusion criteria risk of bias was assessed by comparing each study’s DCM diagnostic criteria with the European Society of Cardiology guidelines.^2^ To assess attrition bias, the number of included participants was compared with the number of identified patients. The results were visualized using traffic light and summary plots produced in RStudio using the ROB1 template from the robvis package.^19–21^

### UK Biobank Sex-Specific Expression of Genotypic and Phenotypic Traits

We also examined whether the unbalanced sex ratio observed in DCM cases could, in part, be driven by use of non–sex-specific diagnostic imaging criteria. We evaluated 47 549 participants from the UK Biobank imaging substudy with cardiac magnetic resonance imaging (CMR) and available whole-exome sequencing. CMR cine images were segmented using an automated deep-learning network^22–24^ to obtain measurements of left ventricular wall thickness and mass, and biventricular volumes. In line with the definition of genetic DCM in the meta-analysis, whole-exome sequencing data were analyzed to identify carriers of variants in 12 definitive or strong evidence DCM genes (Table S1), which would be classified as pathogenic or likely pathogenic if identified in a patient with DCM, using Cardioclassifier and ClinVar (Methods in the Supplemental Material). Hospital Episodes Statistics data obtained from the UK Biobank were used to identify participants who had a record of an ICD-10 (International Classification of Diseases, 10th revision) diagnostic label for DCM. In addition, we derived a novel imaging-phenotype label for DCM, defined using sex-specific cutoff ranges for abnormal indexed left ventricular end-diastolic volume (LVEDV) and left ventricular ejection fraction (LVEF),^25^ in the absence of known valve disease or ischemic heart disease, based on CMR imaging obtained at instance 2 UK Biobank Imaging visit (Methods in the Supplemental Material).

### Ethics

The UK Biobank study recruited 500 000 participants age 40 to 69 years from across the United Kingdom between 2006 and 2010. All participants provided written informed consent for participation in the study, which was approved by the National Research Ethics Service (approval 11/NW/0382). This study was conducted under terms of access approval number 40616.

### Statistical Analysis

Each study’s sex ratio and 95% CI were calculated from published data. In the main analyses, these ratios were logit transformed and pooled on the logit scale (within predefined DCM subgroups) using a random-effects meta-analysis with DerSimonian-Laird weights. The pooled estimates and 95% confidence limits were back-transformed to proportions for presentation. I^2^ estimates were used to assess between-study heterogeneity. Sensitivity analyses used a fixed-effects meta-analysis implemented through a generalized linear mixed model with sex as the outcome variable, a random effect at the study level, and a logit link function.^29-31^ Generalized linear mixed models were also used to assess whether the sex ratio differed by specific characteristics, by including these covariates (measured at a group level) as independent variables in the model (Methods in the Supplemental Material). We compared the sex ratio by whether DCM was genetic or gene-elusive in all patients, and then restricted to the subset of studies that recruited patients with both genetic and gene-elusive DCM. We refer to patients with genetic DCM from these studies as the “reduced genetic DCM cohort.” Statistical analyses were performed in R version 4.2.3 using the packages metafor^19,26–28^ and shapes.^32^

#### Data and Code Availability

Requests for the extracted data and analysis scripts will be considered upon request to the corresponding author.

## RESULTS

The search outputs from each of the databases generated a total of 5787 studies. Removal of duplicates left a total of 3530 studies to be assessed by title and abstract screening, of which 870 progressed to full-text screening. In full-text screening, 692 studies were not relevant, leaving 178 relevant articles (Figure 1). The articles that met the inclusion criteria were then assessed for described cohort duplication, which left 99 studies to be included in the quantitative analysis^10,33–130^ (Figure 1 and Table S3). The studies included patients recruited between 1970 and 2021 and included cohorts ranging from 100^72^ to 4143 participants^73^ (Table S4). The reasons for exclusion can be found in the Preferred Reporting Items for Systematic Reviews and Meta-Analyses flow diagram (Figure 1).

### Quality Assessment

The risk of bias within the 99 studies was visualized by both a summary plot and a traffic plot (Figures S3 and S4). All of the risks of bias were found in <15% of the included studies except for the risk of bias due to retrospective enrollment (Figure S3). This risk was identified in 56 of the studies, with the majority of the studies failing to report whether their described cohorts were consecutively enrolled^34,35,37,38,41,48,49,52,61–63,65,66,71,82,83,85,86,93,97,101,106,111–113,115–121,126,128,129^ or not providing the method of cohort assembly.^36,45,54,55,60,64,72,76,78,81,84,87,89,90,102,105,109,114,123,124,130^ The influence of retrospective enrollment bias on the pooled logit proportion of female participants was found to not be statistically significant for any of the described 4 cohorts (*P*>0.05); therefore, no articles were eliminated due to a high risk of bias (Table S5). All 99 studies were included in the meta-analysis.

### Generation of DCM Subtype Cohorts

The details of the DCM cohorts are presented in Table 1. All of the patients from the 99 studies were included in the general DCM cohort, which comprised 37 525 patients with DCM. Of these studies, 23 described patients with DCM who had received some form of genetic testing.^10,33,37,40,42–44,51,56,75,78,80,84,86,88,97,99,107,108,111,113,120,126^ The genetic testing methodologies used ranged from single gene to large gene panels. The singular DCM-associated genes that had individual studies included *DES*,^*75*^ *RBM20*,^*88*^ *MYH7*,^*86,113*^ and *TTN*.^*10,^33^,^37^,^78^,111*^ The remaining studies used gene panels, which ranged in size from 10^84^ to 183 genes.^120^ These studies generated a genetic DCM cohort that comprised 2069 patients. Out of the studies that performed genetic testing, 10 reported the clinical details of the patients who had not had a pathogenic variant identified and made up the gene-elusive DCM cohort.^40,42–44,78,84,107,108,120,126^ This cohort contained 2020 patients.

**Table 1.**
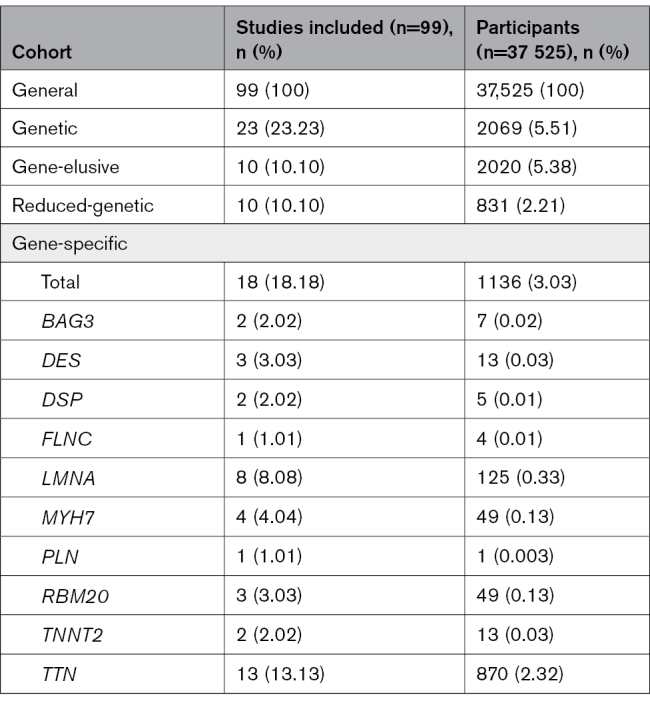
Included Studies and Participants for the Dilated Cardiomyopathy Cohorts

Due to the use of gene panels, patients with DCM with variants within genes that were not included in the 12 predefined DCM genes were included in the genetic DCM cohort. To assess the influence of these patients, the sex ratio data were extracted at the gene-specific level to generate the gene-specific DCM cohort. Only 10 of the 12 DCM-related genes had sex ratio data that could be extracted, excluding *TNNC1* and *SCN5A*. The patients included in this cohort came from 18 of the 23 genetic DCM studies and included a total of 1136 patients (Table 1 and Table S6).^10,33,37,40,42,43,51,56,75,78,80,84,86,88,107,111,113,126^

### DCM Subcohort Proportion Determination and Comparison

#### All-Cause DCM

**Figure 2.**
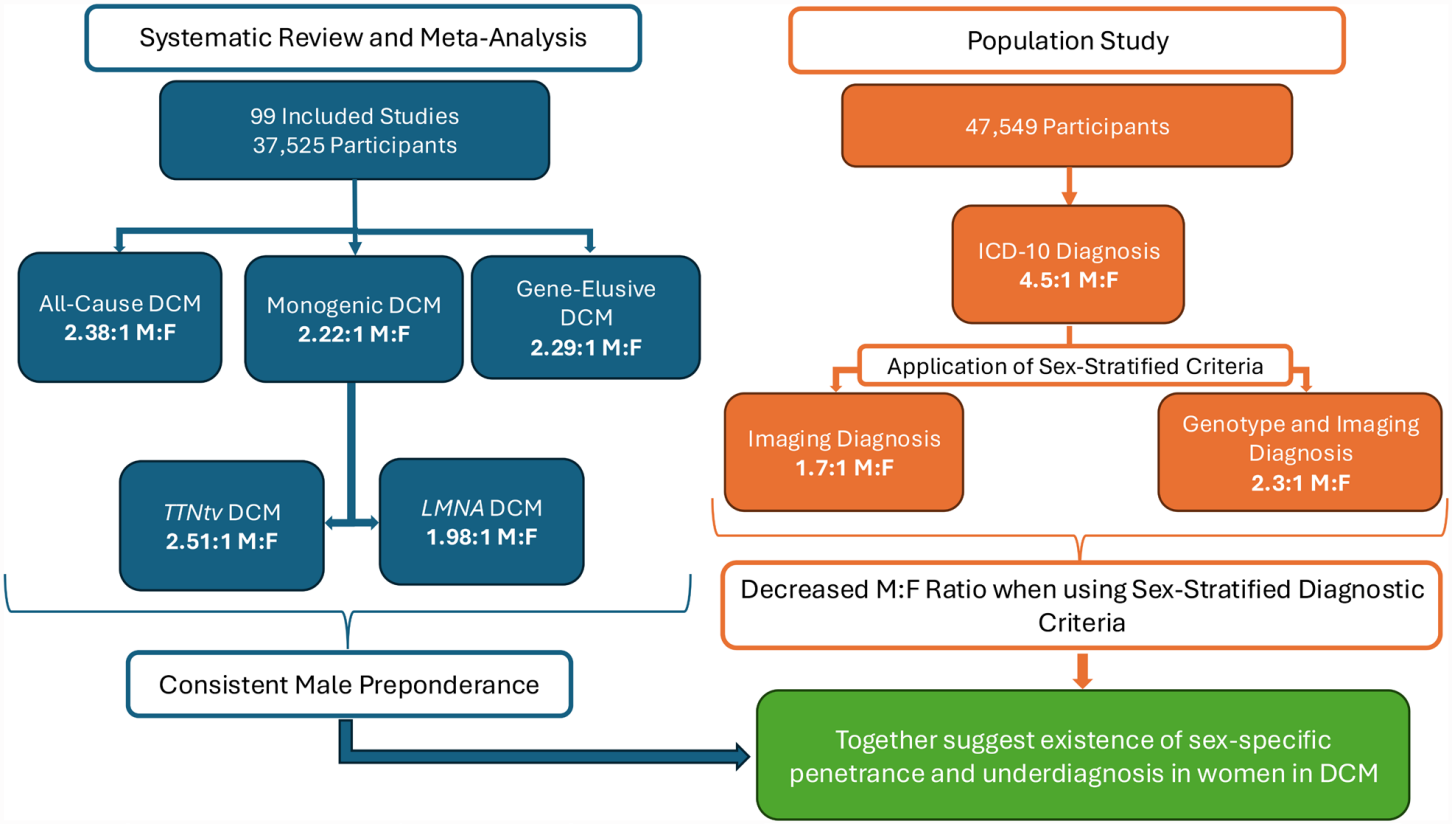
**Summary results illustration.** This figure displays the main summary results found in this investigation. The determined male to female ratios (M:F) are presented for both the systematic review and meta-analysis and the population study. Multiple sources of evidence demonstrate a male preponderance in dilated cardiomyopathy (DCM), which is partly mitigated by applying sex-specific diagnostic criteria. ICD-10 indicates International Classification of Diseases, 10th revision; and *TTN*tv, titin-truncating variant.

**Figure 3.**
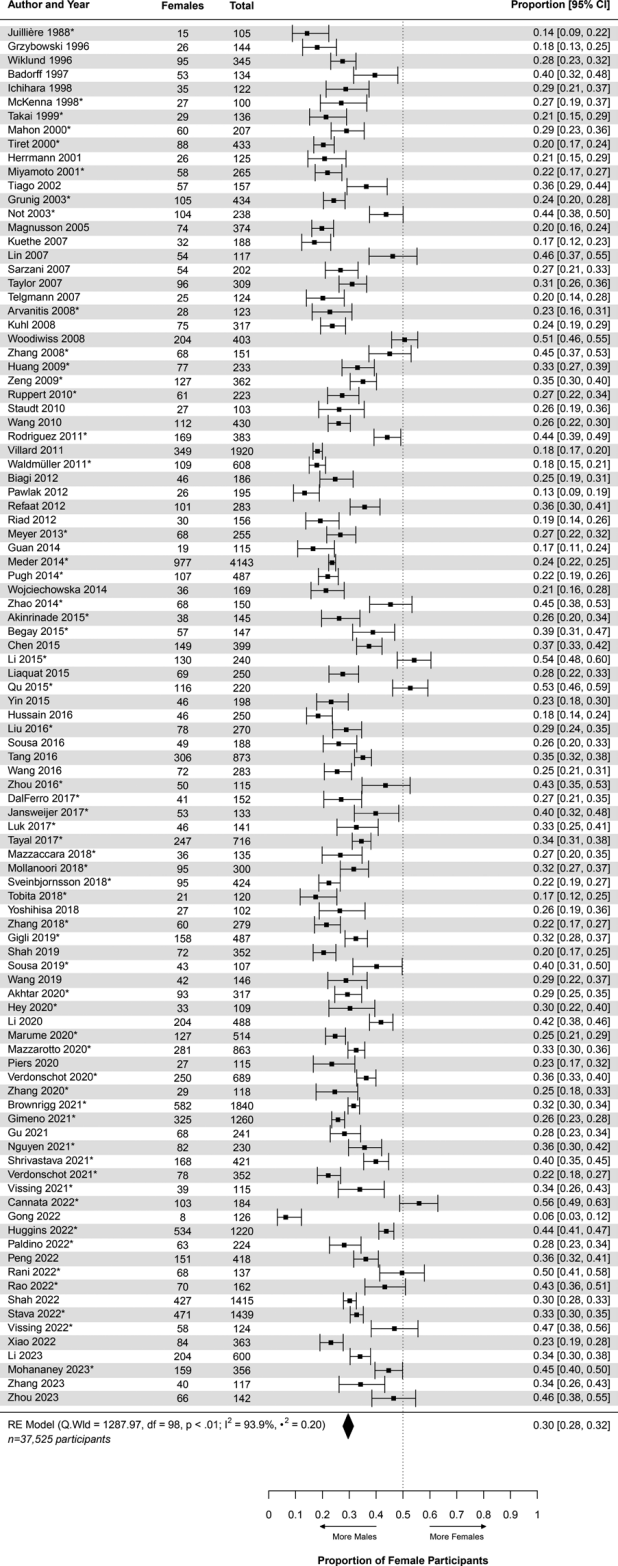
**Forest plot of general dilated cardiomyopathy cohort.** This figure displays the proportion of female patients with dilated cardiomyopathy and the 95% CI for each of the included studies along with the pooled estimate of 0.30 (95% CI, 0.28–0.32; Table 2). The diamond corresponds to the pooled proportion at its center and the diamond’s width represents the 95% CI. The heterogeneity estimates are provided. The asterisk next to the study identifier indicates familial patients are included within that study’s population. RE indicates random effects.

For the all-cause DCM cohort, the pooled proportion of female participants was 0.30 (95% CI, 0.28–0.32; Table 2 and Table S8). This proportion was insensitive to the choice of fixed- or random-effects meta-analysis (Table S7). This proportion informed an M:F of 2.38:1 (Table 2 and Figure 2). The included individual studies (n=99) proportions ranged from 0.06 (95% CI, 0.03–0.12)^45^ to 0.56 (95% CI, 0.49–0.63),^40^ with 5 (5.05%) of the studies having proportions ≥0.5^40,60,85,86,119^ (Figure 3).

**Table 2.**
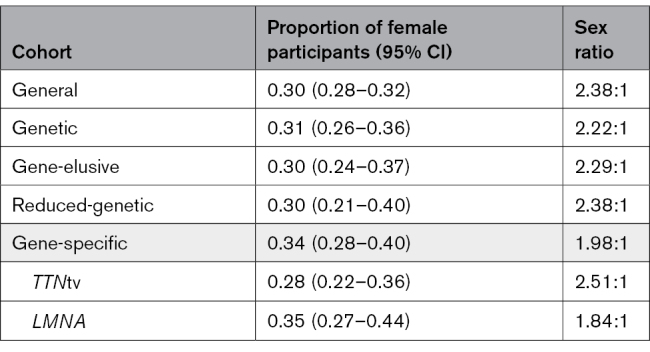
Pooled Proportion of Female Participants for Each Described Cohort

#### Genetic DCM

**Figure 4.**
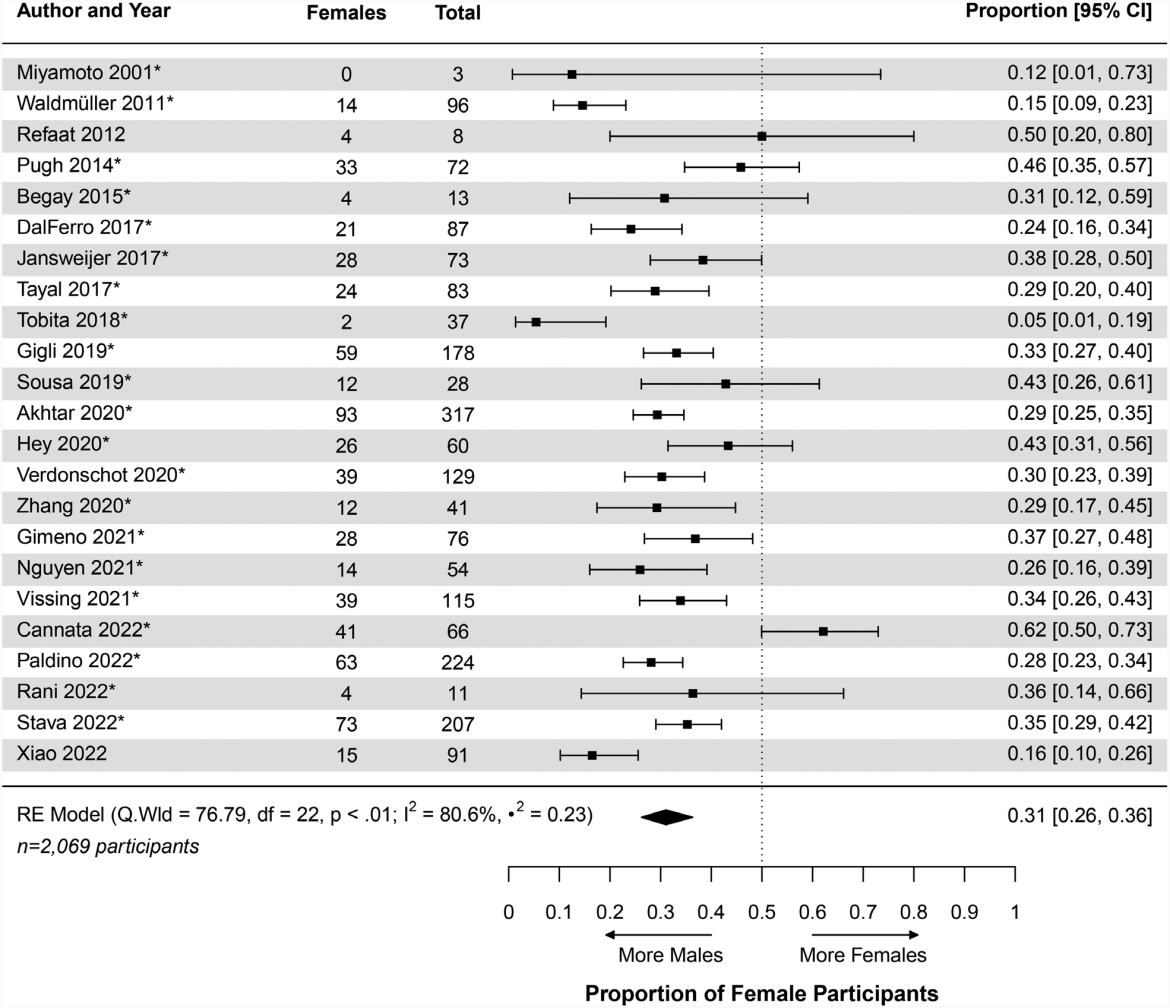
**Forest plot of monogenic dilated cardiomyopathy.** This figure displays the individual study and pooled proportions of female patients for the genetic dilated cardiomyopathy cohort. The genetic cohort proportion was 0.31 (95% CI, 0.26–0.36; Table 2). The asterisk indicates the study includes familial patients with dilated cardiomyopathy. The diamond’s center corresponds to the pooled proportion and the width represents the 95% CI. RE indicates random effects.

The pooled proportion of female participants within the genetic DCM cohort was 0.31 (95% CI, 0.26–0.36; Table 2 and Table S9). This informed an M:F of 2.22:1 (Table 2 and Figure 2). The included individual studies (n=23) proportions were heterogeneous (likelihood ratio test *P*<0.0001; I^2^=80.6%; Table S9) and ranged from 0.05 (95% CI, 0.01–0.19)^107^ to 0.62 (95% CI, 0.50–0.73)^40^ (Figure 4). Two of the studies (8.70%) had proportions ≥0.5.^40,88^ On metaregression analysis, there was no significant difference between the proportion of female patients for the general and the genetic DCM cohorts (*P*=0.57; Table 3).

**Table 3.**
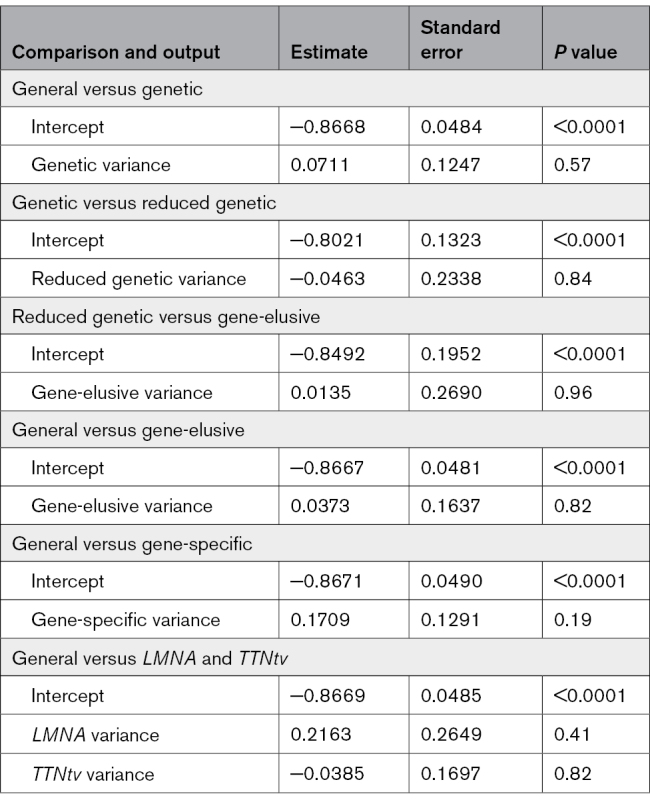
Comparative Metaregression Results

#### Gene-Elusive DCM

**Figure 5.**
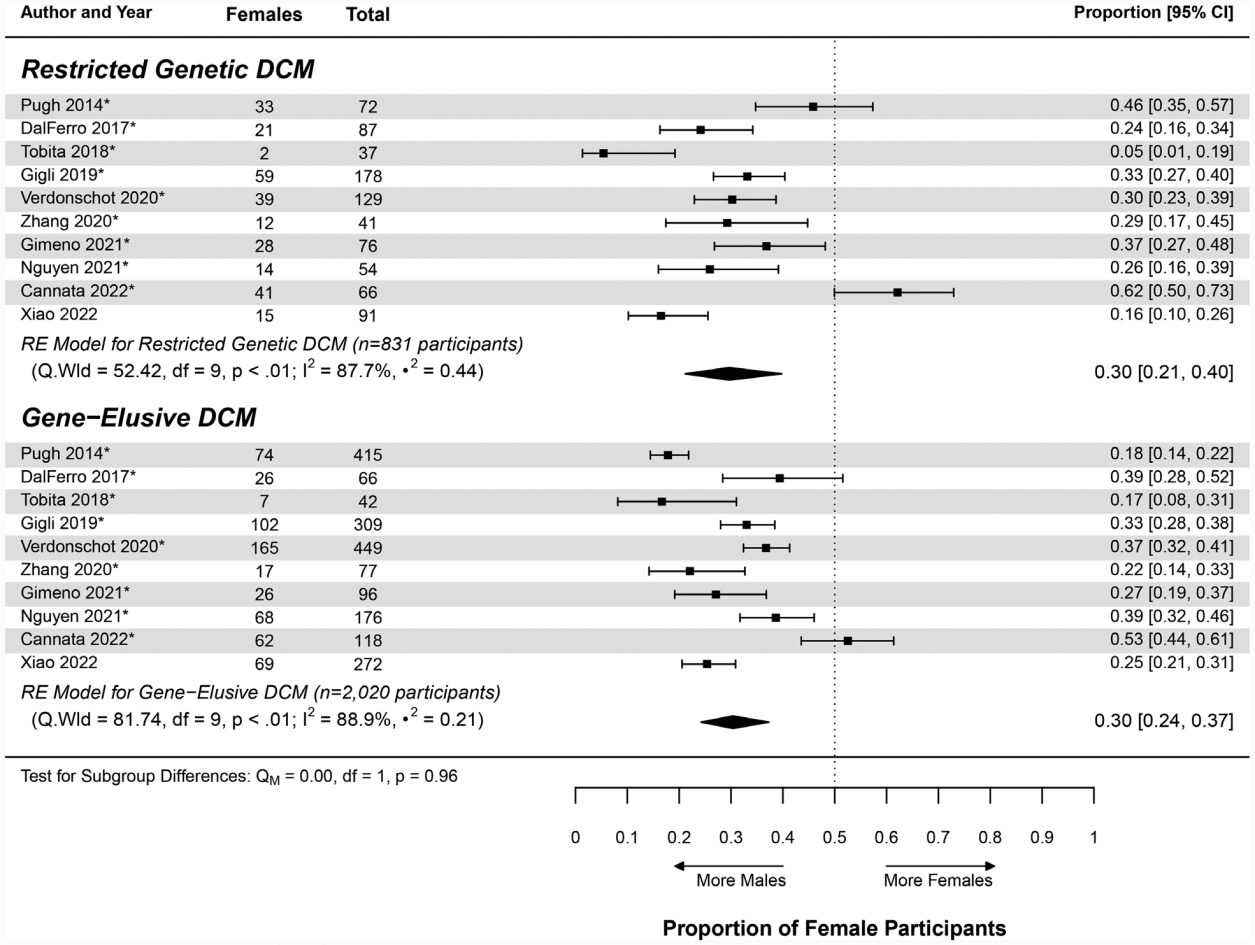
**Forest plot of reduced genetic and gene-elusive cohorts.** This figure displays the proportions for the genetic and gene-elusive dilated cardiomyopathy (DCM) cohorts derived from the 10 studies that described both populations. The individual study and pooled proportions are provided. The gene-elusive cohort proportion was 0.30 (95% CI, 0.24–0.37) and the reduced genetic proportion was 0.30 (95% CI, 0.21–0.40; Table 2). The asterisk next to the study’s identifier indicates the inclusion of familial patients with DCM. The diamond’s center represents the pooled proportion, and the width represents the 95% CI. RE indicates random effects.

For the gene-elusive DCM cohort, the pooled proportion of female participants was 0.30 (95% CI, 0.24–0.37; Table 2, Table S10, and Figure 5). This proportion informed a sex ratio of 2.29:1 (Table 2 and Figure 2). The contributing individual studies (n=10) had proportions that ranged from 0.17 (95% CI, 0.08–0.31)^107^ to 0.53 (95% CI, 0.44–0.61)^40^ (Figure 5). One study (10.00%) had a proportion >0.5^40^ and, as seen with the genetic DCM cohort, the data were heterogeneous (likelihood ratio test *P*<0.0001; I^2^=89.0%; Table S10). The proportion of female participants in the gene-elusive DCM cohort was not significantly different from the general DCM proportion (*P*=0.82; Table 3).

To compare the gene-elusive and genetic cohorts, the genetic cohort was reduced to only include the participants from the studies that were also included in the gene-elusive pooled proportion (n=10 studies).^40,42–44,78,84,107,108,120,126^ This reduced genetic DCM cohort was found to have a pooled proportion of female participants of 0.30 (95% CI, 0.21–0.40; Table 2, Figure 5, and Table S11). This reduced genetic DCM cohort proportion was found to not significantly differ from the total genetic DCM cohort proportion (*P*=0.84; Table 3). Again, the proportion of female patients in the gene-elusive DCM cohort and the reduced genetic DCM cohort did not differ significantly (*P*=0.96; Table 3).

#### Gene-Specific DCM

**Figure 6.**
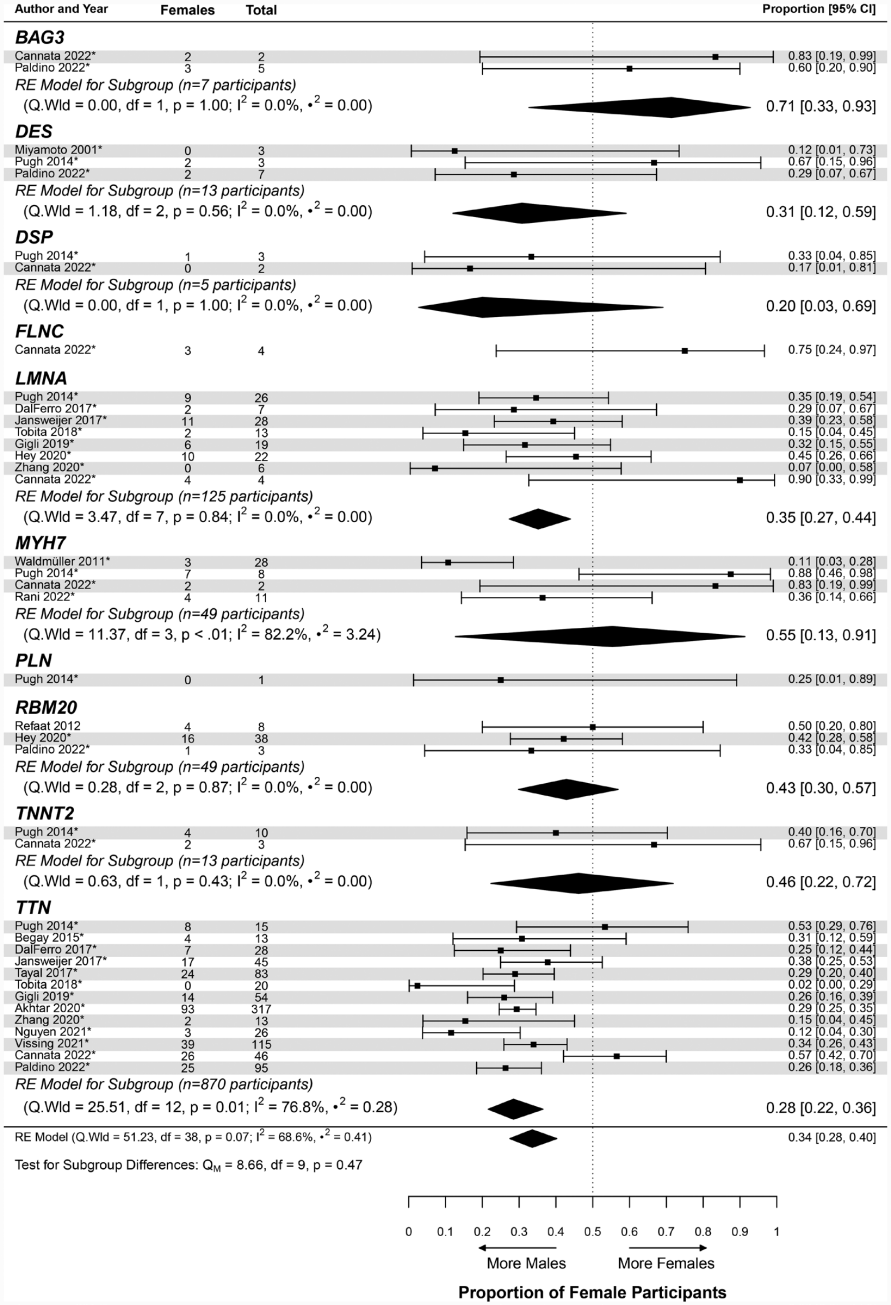
**Forest plot of the gene-specific dilated cardiomyopathy cohort.** This figure displays the inverse-logit proportions of female participants with dilated cardiomyopathy (DCM) of the autosomal dominant genetic DCM cohort. The pooled proportion was found to be 0.34 (95% CI, 0.28–0.40; Table 2). The data are divided by gene, with certain studies falling under multiple genes. The total number of study participants included in each gene group is provided. The asterisk next to the study author and year indicates familial patients with DCM are present within the study’s cohort. The center of the diamond indicates the pooled proportion, and the width represents the 95% CI. RE indicates random effects.

The gene-specific DCM cohort had a pooled proportion of female patients of 0.34 (95% CI, 0.28–0.40; Table 2 and Table S12). This informed a sex ratio of 1.98:1 for the gene-specific cohort (Table 2). The proportions calculated for the included studies ranged from 0.02 (95% CI, 0.00–0.29)^107^ to 0.90 (95% CI, 0.33–0.99)^40^ (Figure 6) and were found to be statistically heterogeneous (likelihood ratio test *P*<0.0001; I^2^=68.6%; Table S12). For the assessed DCM-associated genes, the calculated pooled proportions ranged from 0.20 (95% CI, 0.03–0.69) for *DSP* to 0.71 (95% CI, 0.33–0.93) for *BAG3* (Figure 6). The proportion was the largest for *FLNC* at 0.75 (95% CI, 0.24–0.97), but this was determined from a single study (Figure 6). Overall, cohorts with *TTN*tv and *LMNA* variants were enriched for male patients, with the gene cohorts generating female proportions of 0.28 (95% CI, 0.22–0.36) and 0.35 (95% CI, 0.27–0.44), respectively (Table 2, Figure 6, and Table S13). Apart from *TTN*tv and *LMNA*, there were insufficient data to interpret the sex ratios in other genes of interest, with wide-ranging CIs due to smaller cohort sizes. Despite the gene-specific DCM cohort having a higher pooled proportion, when compared with the general DCM cohort by a metaregression, the difference was not statistically significant (*P*=0.18; Table 3). A meta-analysis assessing differences between the pooled proportions for the individual genes could not be completed because of the highly variable and small cohort sizes.

#### Influence of Ancestry

Of the 99 articles included in this study, 42 provided some details on self-reported race or ethnicity. The pooled proportion of female participants in the European cohort compared with the general DCM cohort all-ethnicities group was lower, indicating that if a person has DCM and is of European ethnicity, they are more likely to be male than in the other assessed ethnicities (proportion of female participants, 0.25 [95% CI, 0.21–0.29]; data from 9 studies, metaregression *P*=0.02; Results in the Supplemental Material and Table S14). However, the pooled proportion of female participants did not differ between European ethnicity and the all-ethnicity groups in the genetic, gene-elusive, or gene-specific DCM cohorts (Results in the Supplemental Material and Table S14). There were no significant differences comparing other ethnicity groups, although available sex- and ethnicity-stratified data were limited (Results in the Supplemental Material).

#### Sensitivity Analyses to Identify Factors Contributing to Heterogeneity

##### Genetic Testing Methodology

The methodology of genetic testing was not consistent across the studies, comprising next-generation, Sanger, whole-exome, and other sequencing. Metaregression showed that the method of genetic testing did not have a significant influence on the proportion of female participants across the 4 DCM cohorts and therefore the results of this study (Results in the Supplemental Material and Table S15).

##### Time Period of Diagnosis

Studies were included in the meta-analysis if a diagnosis of DCM had been made by the study investigators. A revision for the clinical diagnostic criteria of DCM by the European Society of Cardiology working group was published in 2016,^2^ to a broader definition of DCM that does not mandate a specific LVEF threshold. Metaregression showed that the time period of recruitment (pre versus post 2016) influenced the results within the genetic and gene-specific subgroups only (Results in the Supplemental Material and Table S16). For both genetic and gene-specific cohorts, a person with genetic or gene-specific DCM was more likely to be male if they were diagnosed after 2016.

##### Age

In sensitivity analysis to evaluate the effect of age on our results, we compared the observed proportions across the 4 DCM cohorts by age, stratified by those older or younger than 50 years (the median age at diagnosis of DCM^131^; Results in the Supplemental Material and Table S17). Metaregression showed that the proportion of female participants was similar across the groups when stratified by age (Results in the Supplemental Material and Table S17). This indicates that age does not appear to be a significant factor when assessing why more male patients are diagnosed with genetic DCM.

##### Geographic Location

We were not able to undertake a country-specific analysis, so studies were grouped into regions of Europe, North America, Asia, and other. Metaregression showed that in studies comprising cohorts from Asia, participants were more likely to be male in the genetic and gene-specific cohorts, compared with the proportions from all geographic regions combined (Results in the Supplemental Material and Table S18). In the general DCM cohort, studies that comprised participants from North America showed a higher proportion of female patients compared with the proportions from all geographic regions combined (Results in the Supplemental Material and Table S18). Heterogeneity decreased in the genetic and gene-specific cohorts upon region-specific analyses.

### UK Biobank Sex-Specific Expression of Genotypic and Phenotypic Traits

We sought to examine whether the imbalanced sex ratio observed in DCM cases could, in part, be driven by heterogeneity in the diagnostic criteria, and specifically the use of non–sex-specific diagnostic imaging criteria. Of 47 549 UK Biobank participants with available data (chamber volumes and ejection fraction) from CMR, 55 (0.12%) had an ICD-10 diagnosis of DCM, 10 of whom were female (18.18%; M:F 4.5:1; Figure 2). When a sex-specific imaging-phenotype label was applied to the cohort, 377 participants (0.79%) were identified as meeting the criteria for phenotypic DCM, 139 of whom were female (36.9%; M:F 1.7:1; Figure 2).

We then applied a genotype-first approach to examine the proportion of male and female variant carriers who manifest DCM defined either by ICD-10 code or by an imaging-defined phenotype: 190 participants (0.4%) were found to be carriers of a pathogenic or likely pathogenic variant (Methods in the Supplemental Material). Of these, 8 (4%) had an ICD-10 diagnosis of DCM, and all 8 were male. When a sex-specific imaging-phenotype label for DCM was used in place of an ICD-10 code, 13 of 190 pathogenic or likely pathogenic variant carriers (7%) were identified, 4 of whom were female (31%; M:F 2.3:1; Figure 2).

## DISCUSSION

This study systematically evaluated the sex ratio for DCM defined by the presence and absence of monogenic variants evaluated as causative. Between all-cause, genetic, and gene-elusive DCM cohorts, a consistent M:F of ≈2.2:1 was observed. The observed sex imbalance partially corrects on implementing sex-specific diagnostic criteria, suggesting that DCM may not be adequately detected in female patients, potentially leading to underdiagnosis. However, the sex imbalance does not fully correct. This supports that DCM may be subject to sex-specific penetrance for those at genetic risk, subject to biologic and environmental factors that increase the risk of DCM diagnosis in male patients in all-cause DCM, or diagnosed more frequently or earlier in men regardless of subgroup.

Diagnostic criteria for DCM are sex agnostic. Increased clinical vigilance is required to diagnose DCM in female patients across the disease spectrum, and the application of sex-specific DCM diagnostic criteria should be considered. A potential explanation for the sex imbalance is that female patients with DCM are underdiagnosed compared with their male counterparts due to conservative diagnostic thresholds. Our interrogation of the UK Biobank data partially validated this observation in the general population. Using sex-stratified imaging criteria and a phenotype-first approach to diagnosis, the male to female imbalance declined from 4.5:1 to 1.7:1. In addition, those with pathogenic or likely pathogenic variants in DCM genes in the UK Biobank who had a diagnosis of DCM were all male, but when sex-stratified imaging criteria were applied, 4 female patients and 1 additional male patient were identified. LVEF is known to be higher in female patients compared with male patients in the healthy population, yet these differences are not accounted for in disease.^132^ In addition, LVEF may not adequately reflect contractile dysfunction in female patients, and the evaluation of sex-specific approaches to DCM diagnosis remains a major unmet need.

The sex-stratified imaging criteria we applied are based on sex-specific reference ranges of CMR indexed values for left ventricular dilation and function.^25^ International guidelines (eg, European Society of Cardiology 2016^2^) recommend diagnosing DCM on the basis of left ventricular dilation and impaired function, with dilation defined as >2 SD from normal corrected to body surface area and sex, and do not recommend a specific LVEF cutoff (eg, <45%). Whereas sex-specific criteria are not widely taken up in clinical practice, they are in line with these clinical guidelines to diagnose DCM. Despite this, sex-specific diagnostic imaging criteria are not applied in clinical practice (eg, the UK Biobank population study demonstrated that more male patients were being diagnosed with DCM on the basis of ICD-10 codes). We showed that if we take a genotype-first approach and then apply sex-specific diagnostic criteria (derived and implemented on the basis of published normal ranges), we could potentially diagnose more women with DCM. The novelty of this work is the demonstration of the potential utility of these sex-specific diagnostic criteria. The AD gene-specific DCM of 1.98:1 M:F could also be explained by genetic and environmental modifiers influencing the penetrance or expressivity of DCM genetic variants in a sex-specific manner. There are a multitude of genes (eg, in the immune system) that are differentially expressed between male and female patients, which could be influencing the phenotypic manifestation of genetic DCM (eg, *TLR7*).^133–135^ In addition, the differentially expressed sex hormones between male and female patients could also affect genetic DCM susceptibility. Female patients experience a unique reproductive lifespan from menarche to menopause, and, for some, during pregnancy and its complications. The average age at onset of DCM is 40 to 50 years, which for women is commonly premenopausal. In vivo studies highlight the protective effects of estrogen on the cardiac phenotype, reducing hypertrophy, cardiac apoptosis, and inflammation.^136^ The premenopausal state may putatively be protective for female patients with at-risk DCM variants, which could be reducing the phenotypic manifestation of DCM or leading to a milder DCM phenotype that is undetected, but this requires further evaluation.

On the contrary, the higher levels of testosterone in male patients could contribute to male genetic DCM susceptibility. One of the major environmental risk factors for DCM is infection, specifically due to its role in the development of myocarditis.^2^ Male individuals are more prone to severe infections because the anti-inflammatory nature of testosterone leads to a reduced immune response.^137^ Furthermore, certain DCM-associated genes, including *BAG3*, *DSP*, and *SCN5A*, have been associated with myocarditis and increased cardiomyopathy predisposition after infection.^4,138^ The combinatorial effect of the environmental and genetic risk factors could be contributing to an increased susceptibility to development of the DCM phenotype or to a more severe phenotype in male patients, leading to the observed male preponderance of genetic DCM. However, the influence of sex-specific environmental factors requires further investigation.

Another potential factor influencing the observed sex ratio could be the variant-carrying gene. The described AD gene-specific cohort is dominated by patients who carry *TTN* and *LMNA* causative DCM variants, with *TTN* alone contributing 77% of the cohort (Table 1). This is not unexpected, as *TTN* is the most common causative gene for DCM.^9^ However, it is possible that DCM attributed to *TTN* variants has a male preponderance, and that the other less-represented genes exhibit different sex ratios that are being masked. It has been reported that *DSP* cardiomyopathy has a female preponderance,^139,140^ but the influence of this gene is not observed here due to the low inclusion of patients with *DSP* variants. Other genes, such as *PLN*, have been reported as having sex ratios much closer to 0.5.^141^ Specific gene studies had significant variance in sample sizes, which prevented a meaningful comparison of the sex ratios for the different included genes and indicates a need for further investigation.

Overall, the meta-analysis found that the sex ratio of AD genetic DCM is not 1:1, but that the sex incidence rate of DCM of ≈2.2:1 M:F remains relatively consistent across the different subtypes. These findings are novel and expand upon previous research by systematically establishing the sex ratio in DCM in an effort to improve our understanding of who is at risk of developing DCM. Conclusively establishing that male patients are at higher risk of getting a DCM diagnosis (and that female patients are at a lower risk) despite an equally prevalent genetic risk sets the foundation for future work evaluating the factors that underpin these sex biases.

### Limitations

This study has directly expanded upon the published literature by determining a systematic novel sex ratio for genetic DCM, giving the ratio increased reliability and generalizability compared with published ratios generated from single or small groups of cohorts, but there are potential limitations to this analysis. It is possible that fewer female patients participated in the included studies, but this possibility is countered by the exclusion of studies with a risk of bias from retrospective enrollment not significantly influencing the pooled proportion (Table S5). Although the influence of retrospective enrollment bias was assessed and determined to not have a significant effect, the inclusion of articles with this bias means the calculated proportion does not represent a fully consecutive cohort. Referral bias may have also contributed to the observed findings; however, given that female patients with DCM have a milder phenotype,^142^ one may have expected more female patients to survive to be referred to specialist academic centers, where many of these cohorts were enrolled; this would have skewed the distribution to favor more female patients. It is equally possible that more severely affected male patients with DCM would be more likely to be referred to specialist centers. Whereas this referral bias may have influenced the results of the meta-analysis, it is unlikely to have affected the population study, where the male preponderance was also replicated. The various meta-analysis cohorts were also determined to have significant levels of heterogeneity, as indicated by the likelihood ratio test *P* value of <0.0001 and the I^2^ values >50% (Tables S8–S12). Although for most types of meta-analyses significant heterogeneity reduces the reliability of the conclusions, it is an expected result for meta-analyses of prevalence values due to temporal and geographic differences between the included studies.^143^ To assess the influence of temporal and geographic differences, metaregressions were performed that compared the proportions of female participants by potential influences. Across potential influencing factors, including ancestry, age, diagnostic criteria across time periods, genetic testing methodology, and geographic location, none of the factors appeared to have had a major influence on the high levels of heterogeneity seen in the described cohorts (I^2^>50%; Tables S14–S18). However, some of the factors appeared to have an influence for certain subgroups, particularly for the genetic and gene-specific subgroups. The analysis found that heterogeneity decreased for Asian ethnicity, post-2016 patient recruitment, and all location subgroups in the gene-specific DCM cohort (I^2^<50%), suggesting that ancestry and geographic location may modify sex-biased penetrance in genetic DCM. A decreased level of heterogeneity was also observed for the gene-elusive cohort when specifically looking at cohorts where the average age was <50 years (I^2^<50%; Table S17). However, these cohorts had smaller numbers of articles included in the groups compared with some of the other subgroups, which could potentially account for the observed reduction in heterogeneity. In addition, not every DCM-associated gene had data that could be individually analyzed, leading the assembled cohort to not fully represent the scope of AD genetic DCM.^9,14^ Metaregressions according to patient characteristics (eg, age, diagnostic criteria) are performed on the basis of study or group-level information. They indicate whether sex ratio differs according to these characteristics at the group level (ie, they are an ecologic analysis and should be interpreted as such). Individual patient meta-analyses would make analyses at the individual level possible and improve statistical power to detect differences in sex ratio according to patient characteristics. The generation of the AD genetic cohort was limited by a shortage of published articles that assessed large DCM patient cohorts on a gene-specific level, which led to the absence of patients with *TNNC1* and *SCN5A* variants in our generated cohort and prevented the comparison of sex ratios at the gene level for many DCM genes (eg, *BAG3*, *DSP*). In addition, the clinical diagnosis criteria for DCM has become progressively broader in recent years (after 2016), with less stringent criteria for left ventricular impairment.^2,144,145^ Older articles will only report participants who had a more severe phenotype, potentially influencing the pooled proportion because a more severe DCM phenotype is reportedly more frequent in male patients,^146,147^ although our sensitivity analyses showed that more male patients were diagnosed with genetic DCM even in the presence of broader diagnostic criteria after 2016. In the population study, sex-specific imaging diagnostic criteria did not fully mitigate the sex imbalance, together suggesting that the male preponderance is not just due to how DCM is diagnosed. Many of the articles also did not report the minimum age requirement for their cohorts, so the cohort used for this investigation cannot be definitively described as >18 years. The main pattern of inheritance for pediatric genetic DCM is autosomal recessive, so the inclusion of these patients could shift the pooled proportion.^148^ The influence of this population was minimized by all the included populations having average ages >18 years. With respect to the population study, whereas efforts have been made to exclude individuals from the UK Biobank data with peripartum or ischemic cardiomyopathy in defining our DCM label, the use of ICD codes is limited in terms of specificity. Peripartum cardiomyopathy will predominantly affect female participants and could have potentially biased our results toward a higher proportion of female patients. Peripartum cardiomyopathy is a distinct clinical entity to DCM, although with a shared genetic predisposition.^149^ We did not enrich our study for peripartum cardiomyopathy and we consistently see male preponderance across subgroups, therefore this is unlikely to account for the observed sex distribution. A proportion of women with DCM present during the peripartum period. It is likely that these women are underrepresented in disease cohorts related to underuse of genetic testing and the previously held beliefs that peripartum cardiomyopathy is an acquired rather than genetic disease.

### Future Directions

To further characterize the sex ratio of genetic DCM, an assessment of the prevalence of pathogenic DCM variants compared with the manifestation of the DCM phenotype in other diverse populations is needed. This could be complemented by taking a genotype-first approach to a systematic review and meta-analysis, as compared with the clinical phenotype-first approach taken in this investigation. A genotype-first or population-ascertained approach may permit a more direct quantification of penetrance, perhaps without the ascertainment bias and influence of sex-biased categorical cut-offs in LVEF and volumes. An equal sex ratio for AD genetic DCM when assessed from a genotype-first approach would implicate underdiagnosis of DCM in female patients as the influencing factor for the unequal sex ratio observed in this investigation. This could indicate that the diagnostic criteria are insufficient for diagnosis in female patients and require reevaluation. Another potential avenue for future research is to compare the genetic architecture of PPCM (genetic testing is underused in PPCM) with the genetic architecture in female patients with DCM to evaluate sex-biased penetrance in DCM-associated genes. Determining why AD genetic DCM is manifesting at different rates for male and female patients would allow clinicians to better predict who is at risk for developing the condition and could potentially allow the condition to be modified, ultimately reducing the burden of DCM on patients and the health care system alike.

## CONCLUSIONS

The results of this investigation demonstrated a consistent ≈2.2:1 M:F for DCM. Even in a population that exclusively contained patients with DCM attributed to AD P/LP variants, the sex ratio was 1.98:1 male to female. This population should be characterized by equal sex prevalence, yet the preponderance of DCM in male patients remained. Sex-stratified imaging diagnosis partly reduces the imbalance but does not eliminate it, suggesting underdiagnosis in female patients may be a contributory factor but not a complete explanation and that there are persistent sex imbalances in DCM disease penetrance. Future studies should be targeted to define the factors underpinning this finding.

## Article Information

### Acknowledgments

This research was conducted using the UK Biobank resource (National Research Ethics Service, 11/NW/0382). This study was conducted under the terms of access of projects 40616 and 47602. The views expressed in this work are those of the authors and not necessarily those of the funders. For the purpose of open access, the authors have applied a creative commons attribution (CC-BY) license to any author held version at acceptance.

### Sources of Funding

This work is supported by the Medical Research Council (grants MR/W023830/1 and MC_UP_1605/13), National Institute for Health Research Imperial Biomedical Research Centre, the British Heart Foundation (grants SP/17/11/32885, PG/19/6/34387, RE/18/4/34215, RG/19/6/34387, FS/IPBSRF/22/27059, RE/24/130023, and CH/P/23/80008), and Sir Jules Thorn Charitable Trust (grant 21JTA). Drs Ware and O’Regan are supported by CureHeart, the British Heart Foundation’s Big Beat Challenge award (grant BBC/F/21/220106). Dr Halliday is supported by the British Heart Foundation (grant FS/ICRF/21/26019) and Rosetrees Trust. Dr de Marvao is supported by the Fetal Medicine Foundation (grant 495237). The funders did not have a role in the study design; in the collection, analysis, or interpretation of data; in the writing of the report; or in the decision to submit the article for publication.

### Disclosures

Dr Tayal is a Freelance Research Editor at the *British Medical Journal*. Dr Tayal has received fees for educational content from Chiesi Medical and is a committee member of the British Cardiovascular Society and Royal Society. Dr Ware has consulted for MyoKardia, Inc, Pfizer, Foresite Labs, Health Lumen, and Tenaya Therapeutics, and receives research support from Bristol Myers Squibb. None of these activities is directly related to the work presented here. Dr O’Regan has consulted for Bayer AG and Bristol Myers-Squibb, is a committee member of the Society for Cardiovascular Magnetic Resonance, and has a patent pending for deep learning cardiac motion analysis for survival prediction in heart failure (Imperial Innovations; assignee; US20210350179A1). Dr Halliday has received honoraria from AstraZeneca. Dr de Marvao is a trustee of the Royal Brompton and Harefield Charity. Dr Lu received payment from OutSee Limited. The other authors declare no competing interests. There are no other relationships or activities that could appear to have influenced the submitted work.

### Supplemental Material

Methods

Results

Tables S1–S18

Figures S1–S4

References 151–152
